# Extensive progressive multifocal leukoencephalopathy as the first manifestation of AIDS

**DOI:** 10.1590/0037-8682-0073-2021

**Published:** 2021-04-12

**Authors:** Lénio Parreira do Couto, Vanessa Barcelos, André Goulart

**Affiliations:** 1 Hospital de Santo Espírito da Ilha Terceira, Serviço de Medicina Interna, Angra do Heroísmo, Portugal.

Progressive multifocal leukoencephalopathy (PML) is an AIDS-defining illness caused by John Cunningham virus (JCV). The most common clinical findings are speech and language disturbances, motor weakness, gait abnormalities, and incoordination. Clinical and radiological features sustain the diagnosis along with detection of JCV in the cerebrospinal fluid (CSF). The majority of HIV patients diagnosed with PML have CD4+ counts <200 cells/µL^1^. 

Brain magnetic resonance imaging (MRI) is the mainstay of radiological evaluation, showing areas of hyperintensity on T2-weighted and fluid-attenuated inversion recovery (FLAIR) images and hypointensity on T1-weighted images. Multiple subcortical lesions are often present in the basal ganglia or thalamus, where myelinated fibers reside^2^. 

**FIGURE 1:**
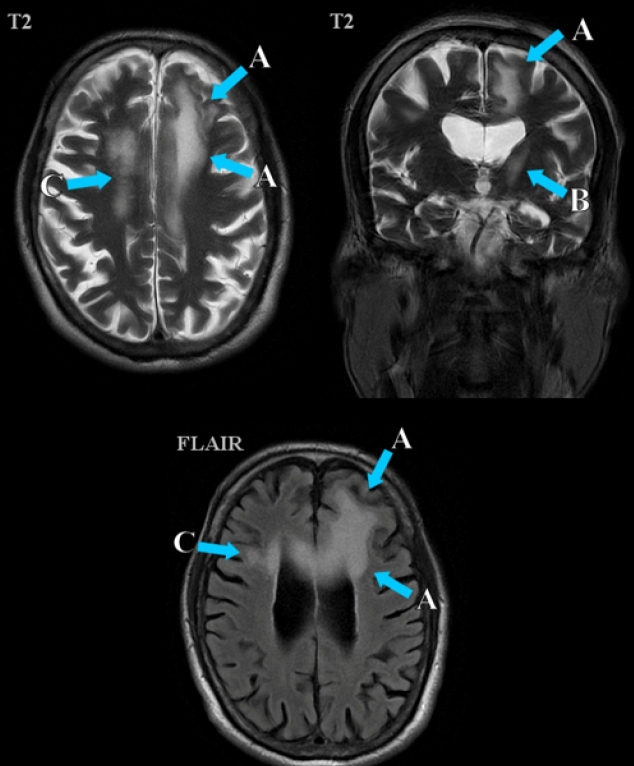
Brain MRI showing an extensive hyperintense lesion on T2 and FLAIR images in the left frontal parasagittal cortico-subcortical **(A)** and striatocapsular regions **(B)** and right frontal subcortical area **(C)**.

A 59-year-old man was admitted for cognitive deterioration, gait difficulty, and urinary incontinence for 3 weeks. Neurological examination revealed severe language and executive function impairments. Infection with HIV-1 was diagnosed 1 year ago, but he had not started antiretroviral therapy, and he was asymptomatic. CD4+ assessment showed 422 cells/mm³. Brain MRI showed evidence of bilateral cortico-subcortical frontal lesions and in the left striatocapsular region, hyperintensity on T2 and FLAIR images with corresponding hypointensity in T1 images, and restriction in diffusion-weighted images (Figure 1). JCV was identified in the CSF, and the diagnosis of PML was made. 

This case illustrates that PML lesions can be extensive and debilitating, even in patients with a high CD4+ count.
